# Cryptotanshinone Inhibits ERα-Dependent and -Independent BCRP Oligomer Formation to Reverse Multidrug Resistance in Breast Cancer

**DOI:** 10.3389/fonc.2021.624811

**Published:** 2021-04-22

**Authors:** Wenting Ni, Hui Fan, Xiuqin Zheng, Fangming Xu, Yuanyuan Wu, Xiaoman Li, Aiyun Wang, Shile Huang, Wenxing Chen, Shijun Wang, Yin Lu

**Affiliations:** ^1^Jiangsu Key Laboratory for Pharmacology and Safety Evaluation of Chinese Materia Medica, School of Pharmacy, Nanjing University of Chinese Medicine, Nanjing, China; ^2^Jiangsu Collaborative Innovation Center of Traditional Chinese Medicine (TCM) Prevention and Treatment of Tumor, Nanjing, China; ^3^Department of Biochemistry and Molecular Biology, Louisiana State University Health Sciences Center, Shreveport, LA, United States; ^4^Shandong Co-Innovation Center of Traditional Chinese Medicine (TCM) Formula, College of Traditional Chinese Medicine, Shandong University of Traditional Chinese Medicine, Jinan, China

**Keywords:** cryptotanshinone, breast cancer resistance protein (BCRP or ABCG2), estrogen receptor α, oligomer formation, multidrug resistance

## Abstract

Both long-term anti-estrogen therapy and estrogen receptor-negative breast cancer contribute to drug resistance, causing poor prognosis in breast cancer patients. Breast cancer resistance protein (BCRP) plays an important role in multidrug resistance. Here, we show that cryptotanshinone (CPT), an anti-estrogen compound, inhibited the oligomer formation of BCRP on the cell membrane, thus blocking its efflux function. The inhibitory effect of CPT on BCRP was dependent on the expression level of estrogen receptor α (ERα) in ERα-positive breast cancer cells. Furthermore, ERα-negative breast cancer cells with high expression of BCRP were also sensitive to CPT because CPT was able to bind to BCRP and inhibit its oligomer formation on the cell membrane, suggesting that the high level of BCRP expression is crucial for CPT to reverse drug resistance. The combination of CPT and chemotherapeutic agents displayed enhanced anticancer effects. The results suggest that CPT is a novel BCRP inhibitor *via* blocking the oligomer formation of BCRP on the cell membrane. CPT is able to inhibit the activity of BCRP in an ERα-dependent and -independent manner, sensitizing breast cancer cells to chemotherapy.

## Background

Chemotherapy is one of the foremost approaches to treat cancer, but the occurrence of multidrug resistance (MDR) has weakened its clinical efficacy (1). MDR happens frequently in breast cancer, especially in estrogen receptor α-positive (ERα+) breast cancer. After receiving hormone therapy such as tamoxifen, about 70% of patients have recurrence of drug resistance in the late stages (2). Recently, many studies have shown a close relationship between the occurrence of breast cancer MDR and the expression of the ATP-binding cassette (ABC) transporter family, especially P-glycoprotein (P-gp/ABCB1), multidrug-resistance-associated protein 1 (MRP1/ABCC1), and the breast cancer resistance proteins (BCRP/ABCG2) (3, 4).

ABCG2, a member of the human ABC transporter superfamily, was known as BCRP, having “drug pump” function (5). BCRP is structurally similar to P-gp and MRP (1), sharing certain homologous sequences (6). However, it still has its own unique conformation, a nucleotide-binding domain (NBD) at the C-terminus and a hydrophobic transmembrane domain (TMD) at the N-terminus, indicating BCRP as a semi-transporter (7). In general, P-gp and MRP1 that have two NBD and multiple TMD structures are called full transporters (8). Semi-transporters are commonly localized in the cytoplasm, but BCRP is the first reported semi-transporter localized on the cell membrane (9). Studies have demonstrated that BCRP is likely to form homodimers, tetramers, dodecamers, and even larger oligomer structures by intramolecular disulfide linkages, which significantly increase the efficiency of external pumping by increasing the formation of the outer channel cavity (10). Thus, inhibiting BCRP or blocking the efflux of therapeutic drugs has been considered a feasible strategy for eliminating the MDR, which boosts the development of BCRP inhibitors (11).

Cryptotanshinone (CPT) is a natural diterpenoid from the plant *Salvia miltiorrhiza*. Since CPT was shown to execute its anticancer action by inhibiting signal transducer and activator of transcription 3 (STAT3) dimerization (12), it has received great attention. We have demonstrated that CPT is able to inhibit the mammalian target of rapamycin (mTOR) signaling (13) and activate the mitogen-activated protein kinase (MAPK) pathways (14), leading to cell death. Of interest, CPT, unlike rapamycin and its derivatives, inhibits mTOR signaling *via* activating the AMP-activated protein kinase (AMPK)–tuberous sclerosis complex 2 (TSC2) axis (15). Most recently, we have observed that the anticancer activity of CPT is related to the status of ERα in breast cancer cells, as MCF7 (ERα-positive) cells are more sensitive to CPT than MDA-MB-231 (ERα-negative) cells (16). Also, MCF-7/ADR, a doxorubicin (DOX)-induced multidrug-resistant cell line, is also sensitive to CPT, and CPT is able to distinctly enhance the inhibitory effect of tamoxifen on MCF-7/ADR (16). MCF-7/ADR cell line is characterized by a high expression of ABC protein family and negative ERα expression induced by DOX to acquire MDR. Therefore, we hypothesized that CPT may target BCRP to reverse the MDR.

In this study, we, for the first time, showed that CPT could inhibit BCRP by interfering with the oligomer formation of BCRP on the cell membrane in both ERα-positive and -negative breast cancer cells. Our results indicate that CPT is a novel inhibitor of BCRP and has great potential to overcome MDR due to high expression of BCRP in both ERα-positive and -negative breast cancer.

## Materials and Methods

### Chemicals and Reagents

CPT [purity 98%, high-performance liquid chromatography (HPLC), Xian Yuxuan Biotechnology Co., Ltd.], RPMI 1640, Dulbecco's Modified Eagle Medium (DMEM), fetal bovine serum (FBS), Opti MEM medium, trypsin-ethylenediamine tetraacetic acid (EDTA), and penicillin/streptomycin were purchased from Gibco (Grand Island, NY, USA). KO143 was obtained from MCE (Newark, NJ, USA). Mitoxantrone (MX) was brought from Meilunbio (Dalian, Liaoning, China). Rhodamine123 was purchased from Sigma-Aldrich (St. Louis, MO, USA). DOX was obtained from Bairui Biotechnology (Nanjing, China). Goat Anti-Rabbit IgG H&L fluorescein isothiocyanate (FITC) was from Abcam (Cambridge, UK). MTS and bovine serum albumin (BSA) were purchased from Biosharp (Hefei, Anhui, China), while radioimmunoprecipitation assay (RIPA) and phenylmethylsulfonyl fluoride (PMSF) were from Dingguo Biotechnology (Beijing, China).

### Cell Culture

Human breast cancer cells (MCF-7 and MDA-MB-231) were obtained from American Type Culture Collection (Manassas, VA, USA). MCF-7 cells were cultured in RPMI 1640 with 10% FBS, and MDA-MB-231 cells were cultured in DMEM with 10% FBS. DOX multidrug-resistant cell line MCF-7/ADR cells were purchased from Nanjing BERKE Biology (Nanjing, China). MCF-7/ADR cells were cultured in RPMI 1640 with 10% FBS and 1.25 μg/ml DOX. All cell lines were cultured in a humid incubator (37°C and 5% CO_2_).

### Cell Viability Assay

MCF-7 cells, MDA-MB-231 cells, and MCF-7/ADR cells were seeded in a 96-well plate at a density of 1 × 10^4^/well. After treating with agents, one solution reagent (MTS, 1:10 dilution in serum-free medium, Promega) was added and incubated at 37°C for 4 h. Finally, the cell viability was evaluated through measuring the optical density (OD) at 490 nm using the BioTek Synergy2 microplate reader (BioTek Instruments, VT, USA).

### High-Performance Liquid Chromatography Analysis

Cell lysates were prepared in the extraction buffer [containing methanol:water (1:1, v/v)] in the cold room for 15 min, followed by scraping and centrifuging at 17,000 g for 10 min. The concentration of CPT in the lysates was measured using HPLC (Waters E2695). Samples were injected into a 4.6 mm × 250 mm Stable Bond column (ZORBAX Eclipse Plus C18; Agilent, CA, USA). The chromatography was run starting with 45% solution A (methanol) and 55% solution B (H_2_O), and the volume of solution A was raised to 50%, 90%, and 100% at 10, 30, and 35 min, respectively. Finally, 45% solution A was used at 45 min and until the end of the assay. Data were collected and analyzed by Analyst Software (AB Sciex).

### Molecular Docking Assay

The three-dimensional structures of CPT and MX were obtained from PubChem Compound database (https://www.ncbi.nlm.nih.gov/pccompound/). Meanwhile, the structure of BCRP/ABCG2 [Protein Data Bank (PDB) ID: 6FFC with resolution of 3.56 Å] was retrieved from the Research Collaborator for Structural Bioinformatics PDB (Anonymous, www.rcsb.org). The molecular docking between the two compounds and BCRP/ABCG2 was evaluated by Discovery Studio (DS) 3.5 using the CDOCKER Protocol under the protein–ligand interaction section after preparing the protein and ligands. The poses were scored by CDOCKER interaction energy, and the binding sites were also shown.

### Plasmids and Transient Transfection

The ERα shRNA (sense: 5′-GATCCCGCTACTGTTTGCTCCTAACCTCGAGGTTAGGAGCAAACAGTAGCTTTTTGGAT-3′; Antisense: 3′-AGCTATCCAAAAAGCTACTGTTTGCTCCTAACCTCGAGGTTAGGAGCAAACAGTAGCGG-5′) (16) was synthesized by Genechem (Shanghai, China). MCF-7 cells were planted in six-well plates at a density of 3 × 10^5^ cells/well. The ERα shRNA plasmid (888 ng/μl) was diluted in Opti-MEM (100 μl) and then mixed with Lipofectamine 2000 reagent (Life Technologies, NY, USA). After 6-h transfection, culture medium was changed to normal medium and sequentially incubated in 37°C for 16 h. The Con-shRNA (535 ng/μl) was used as a negative control.

### Mitoxantrone/Rhodamine 123/Doxorubicin/Topotecan Efflux Experiment

The MX/rhodamine 123 (Rh123)/DOX/topotecan (TOPO) efflux experiment was performed as described (17). Briefly, breast cancer cells (3 × 10^5^ cells per well) were seeded in six-well plates and incubated overnight. At about 80% confluence, the cells were with CPT for 8 h. Then, the cells were collected by centrifugation in 2-ml tubes, and each tube was added with 1 ml of serum-free medium to homogenize the cells. All the cells except the blank group were added with the corresponding compounds and incubated in the dark under 37°C for 30 min (the MX-positive group was treated with KO143 10 μmol/L for 15 min in advance). Next, all cells were centrifuged (1,500 rpm, 4°C, 5 min), and the supernatants were discarded. The cells were washed with pre-cooled PBS twice. Finally, the cells were resuspended in 400 μl pre-cooled PBS. The fluorescence accumulation of MX/Rh123/DOX/TOPO is detected with a BD Accuri C6 Flow Cytometer (Becton, Dickinson and Company, NY, USA). The detection channel was FL-4/FL-1/FL-2. The wavelength of Ex/Em for MX/Rh123/DOX/TOPO is, respectively, 488/660 nm, 488/525 nm, 488/575 nm, and 488/525 nm.

### Non-Reducing Gradient Gel Electrophoresis

The non-reducing gradient gel electrophoresis was performed as described (18). Membrane and cytoplasmic proteins were extracted as described in *Extraction of Cell Membrane and Cytoplasmic Proteins*. The protein samples were denatured with a loading buffer containing no reducing agents such as dithiothreitol (DTT) or 2-mercaptoethanol (2-ME). Samples were boiled at 100°C for 15 min. The remaining steps were essentially identical to those in Western blotting. When detecting the BCRP polymer, membrane proteins were separated by 6% Tris-glycine sodium dodecyl sulfate-polyacrylamide gel electrophoresis (SDS-PAGE), and a multicolor broad range protein ladder ranging from 10 to 260 kDa (Thermo Scientific, Waltham, MA, USA) was used.

### Fluorescence Resonance Energy Transfer Microscopy Imaging

The fluorescence resonance energy transfer (FRET) was performed as described (19). The pCFP-ABCG2 and pYFP-ABCG2 plasmids were generously provided by Jun Wang (Shanghai Institute of Materia Medica, Chinese Academy of Sciences, Shanghai, China). Cells were seeded in a 35-mm confocal culture dish, and when the cell confluence reached 70%, the plasmid transfection was performed following the protocol provided by the manufacturer (Invitrogen, NY, USA). Plasmid pCFP-ABCG2 (191.3 ng/μl), pYFP-ABCG2 (399.3 ng/μl), and 3 μl Lipofectamine 2000 reagent (Life Technologies, NY, USA) were, respectively, mixed with 50 μl Opti-MEM medium. After incubating for 5 min at room temperature, the above two mixtures were lightly mixed and cultured for 10 min at room temperature. Finally, the 100 μl mixture was added dropwise to 1 ml serum-free medium, and 100 μl FBS was added 6 h later. After incubation for 16 h at 37°C, CPT was added. Finally, the living cell FRET images were collected under an inverted fluorescence microscope (Leica Microsystems, Solms, Germany) and analyzed by ImageJ software.

### Immunofluorescence Staining

Cells were plated on glass coverslips in six-well plates and then treated with corresponding compounds. The cell membrane was stained with 10 μmol/L DiI (Beyotime Biotechnology, Shanghai, China) for 10 min at 37°C. Then, cells were fixed in 4% paraformaldehyde for 20 min and blocked in BSA (1% BSA dissolved in PBS). The cells were incubated with the corresponding primary antibody (1:100 dilution) overnight at 4°C, followed by incubation with goat anti-rabbit IgG H&L FITC (1:1,000 dilution) for 2 h and Hoechst nuclear dye for 10 min in the dark. The images were obtained from a laser scanning confocal microscope (Leica TCS SP5 X, Solms, Germany).

### Extraction of Cell Membrane and Cytoplasmic Proteins

Cell membrane protein and cytoplasmic protein were extracted according to the protocol provided by the manufacturer (Beyotime Biotechnology, Shanghai, China). In brief, cells were seeded into 150-mm culture dishes (Lab services, Waltham, MA, USA) and treated with compounds when the cell coverage area reached about 80%. After treatment for 8 h, the cells were washed once with ice PBS and were then collected with a scraper, followed by centrifuging (4°C, 600 g, 5 min). The supernatants were discarded, and the cells were resuspended in reagent A containing PMSF (1:100 dilution) and incubated in an ice bath for 10–15 min. Then, the cells were treated with liquid nitrogen, freezing and thawing twice, and centrifuged (4°C, 700 g, 10 min). The supernatants were collected and centrifuged (4°C, 14,000 g, 30 min) to precipitate cell membrane fragments. The supernatants contained cytoplasmic protein. The pellets were resuspended in reagent B and vortexed for 5 s and incubated in an ice bath for 5–10 min. After centrifugation (4°C, 14,000 g, 5 min), the supernatants contained cell membrane protein, which were stored at −80°C.

### Western Blotting Analysis

Western blot was performed and analyzed as described (15). The primary antibodies were used as follows: ERα, BCRP/ABCG2, MDR1/ABCB1 (Cell Signaling Technology, Danvers, MA, USA), and glyceraldehyde 3-phosphate dehydrogenase (GAPDH; Bioworld Technology, MN, USA). ABCC1 (Affinity, MA, USA) HRP-Goat Anti-rabbit IgG (H+L) was purchased from Bioworld Technology Company.

### RNA Isolation and Real-Time PCR

Total RNA was extracted from MCF-7 or MDA-MB-231 cells with TRIzol (Vazyme, Nanjing, China) according to the manufacturer's guidelines and reversely transcribed to cDNA by HiScript® II Reverse Transcriptase (Vazyme, Nanjing, China). Real-time PCR was executed of the ChamQTM SYBR® qPCR Master Mix (Vazyme, Nanjing, China) using Applied Biosystems 7500 Real-Time PCR Systems (Thermo Scientific, Waltham, MA, USA). GAPDH served as a reference control, and mRNA levels were expressed as fold changes after normalizing to GAPDH. The primers (Sangon Biotech, Shanghai, China) used are listed in Supplementary Table 1.

### Statistical Analysis

The results were determined using Student's *t*-test (two-group comparison) and ANOVA test by GraphPad Prism 5.0 software. *P* < 0.05 was considered statistically significant.

## Results

### Intracellular Accumulation of Cryptotanshinone Is Possibly Related to Breast Cancer Resistance Protein

**Figure 1:**
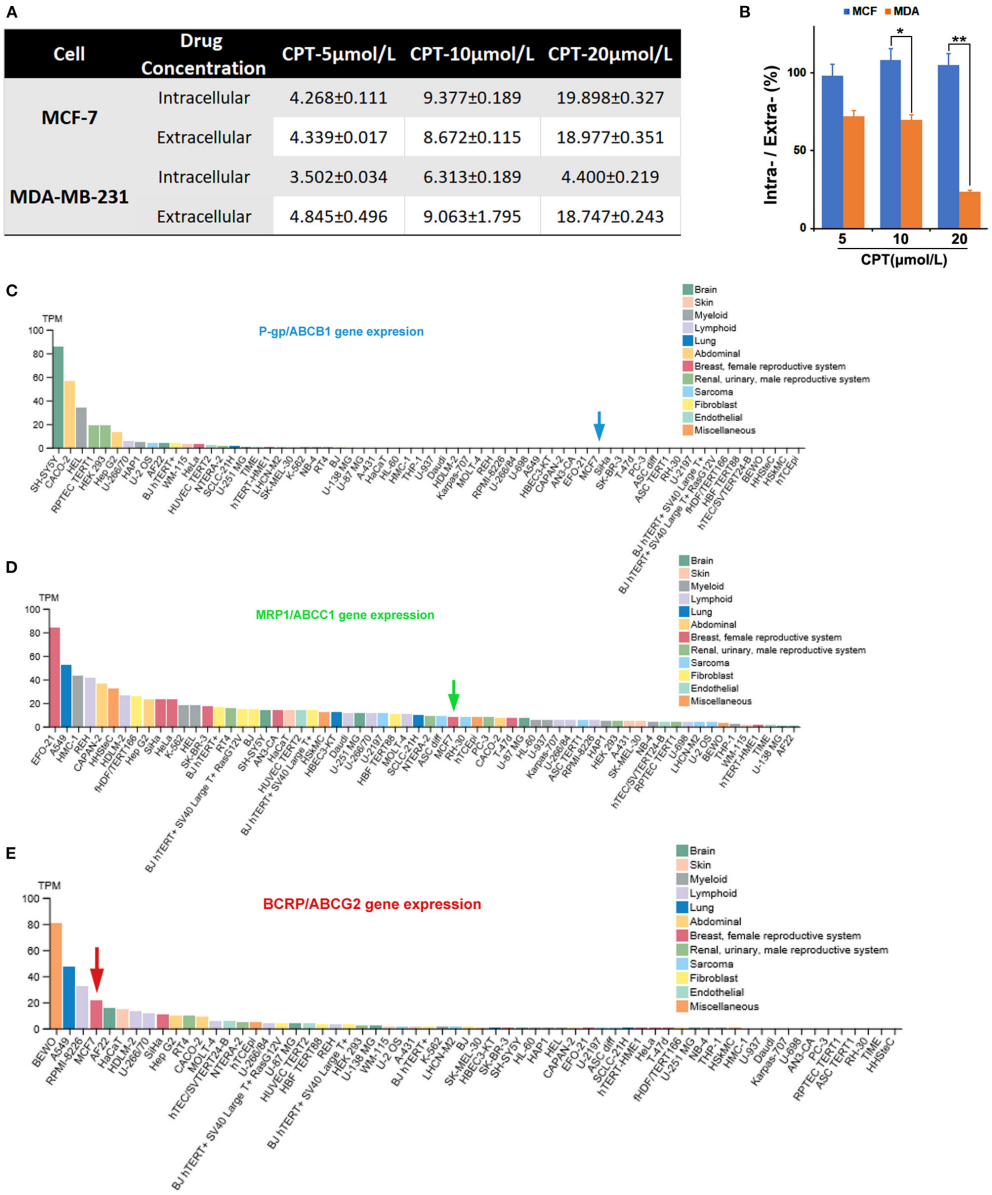
Intracellular accumulation of cryptotanshinone (CPT) in MCF-7 cells is possibly related to breast cancer resistance protein (BCRP). **(A)** The indicated breast cancer cells were treated with CPT (5, 10, 20 μmol/L) for 8 h. Then, the medium was collected, and the intracellular and extracellular CPT concentrations (μmol/L) were detected by high-performance liquid chromatography (HPLC). The data are presented as mean ± SD (*n* = 3). **(B)** Statistical analysis of the ratio of intracellular CPT/extracellular CPT × 100%, *n* = 3, *P < 0.05, **P < 0.01. **(C–E)** The expression of ABCB1/ABCC1/ABCG2 mRNA levels in different cells based on the Human Protein Atlas database analysis.

In order to understand why MCF-7 and MDA-MB-231 cells show different sensitivity to CPT except the status of the ERα, we first detected the intracellular and extracellular CPT levels in the two cell lines by HPLC. As shown in Figure 1A, treatment with CPT dose-dependently increased the intracellular and extracellular levels of CPT robustly and evenly in MCF-7 cells, but not in MDA-MB-231 cells. Of note, the increase in the intracellular CPT level was greatly less than that of the extracellular CPT level in MDA-MB-231 cells. This difference was significant when MDA-MB-231 cells were treated with CPT at 10 or 20 μmol/L (Figure 1B). The result suggests that there is a certain transporter that regulates the intake and pumping process of CPT in the cells. The most studied transporter proteins are ATP-binding transporters, such as P-gp, MRP1, and BCRP (5). We analyzed the expression of these three transporters in many cell lines from multi human tissues with the Human Protein Atlas Database. The results indicated that BCRP/ABCG2 was highly expressed in MCF-7 cells (Figure 1C), whereas P-gp/ABCB1 was almost not expressed and MRP1/ABCC1 was lowly expressed in the cells (Figures 1D,E). Accordingly, we speculated that BCRP may play an important regulatory role in transporting CPT across the membrane.

### Cryptotanshinone Inhibits Efflux Function of Breast Cancer Resistance Protein, Depending on the Expression Level of Estrogen Receptor α in Cells

**Figure 2:**
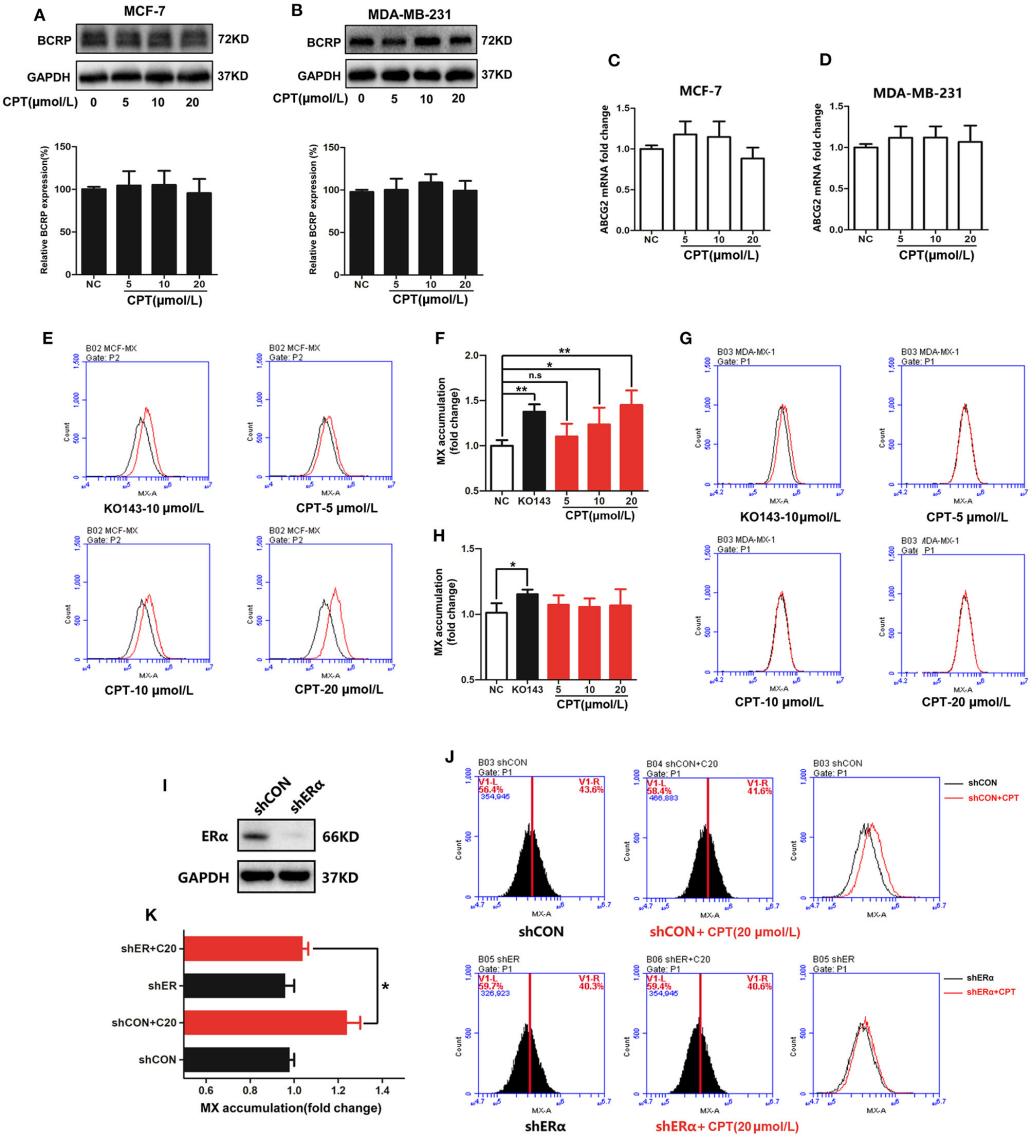
Cryptotanshinone (CPT) inhibits the efflux function of breast cancer resistance protein (BCRP) in MCF-7 cells. Western blot analysis of the protein levels of BCRP in MCF-7 **(A)** and MDA-MB-231 cells **(B)** treated with CPT for 8 h. Q-PCR analysis of the mRNA levels of BCRP in MCF-7 **(C)** and MDA-MB-231 cells **(D)** treated with CPT for 8 h. The mitoxantrone (MX) fluorescence accumulation was detected in MCF-7 **(E)** and MDA-MB-231 cells **(G)** treated with CPT for 8 h by flow cytometry. The fluorescence intensity represents the activity of BCRP efflux. The quantitated results are shown in panels **(F,H)**, respectively, vs. negative control (NC), *n* = 3, *P < 0.05, **P < 0.01. **(I)** MCF-7 cells with shCON or with shERα were, respectively, treated with or without CPT (20 μmol/L) for 8 h and then **(J)** analyzed for MX fluorescence accumulation by flow cytometry. The quantitated results are shown in **(K)**, *n* = 3, *P < 0.05.

The close relationship between CPT and BCRP led us to further explore the specific regulation of CPT on BCRP. Firstly, CPT had no significant effect on the total protein expression of BCRP in both MCF-7 and MDA-MB-231 cells (Figures 2A,B). In addition, CPT did not significantly affect ABCG2 mRNA levels in MCF-7 and MDA-MB-231 cells by q-PCR (Figures 2C,D; Supplementary Table 1). Considering that the most important ability of BCRP is to transport substrates, next we examined whether CPT influences the function of BCRP. For this, we evaluated the efflux function of BCRP through the MX efflux experiment (20). Compared with the positive control Ko143, a selective BCRP inhibitor, the fluorescence peak of CPT-treated group in MCF-7 cells was also significantly shifted to the right (Figures 2E,F). The increased fluorescence abundance of intracellular MX indicated that the efflux function of BCRP was inhibited. However, this effect was not observed in MDA-MB-231 cells (Figures 2G,H). So we wondered if the difference between the two cells might be related to the expression of ERα. To this end, the expression of ERα was silenced in MCF-7 cells, followed by treatment with CPT for 8 h (Figure 2I). Interestingly, knockdown of ERα partially decreased the accumulation of MX induced by CPT treatment (Figures 2J,K). These results indicate that CPT does not affect the cellular protein and mRNA levels of BCRP/ABCG2 but is able to inhibit the efflux function of BCRP in MCF-7 cells, and this effect is closely relevant with the presence of ERα.

### Cryptotanshinone Reduces the Level of Breast Cancer Resistance Protein on the Cell Membrane

**Figure 3:**
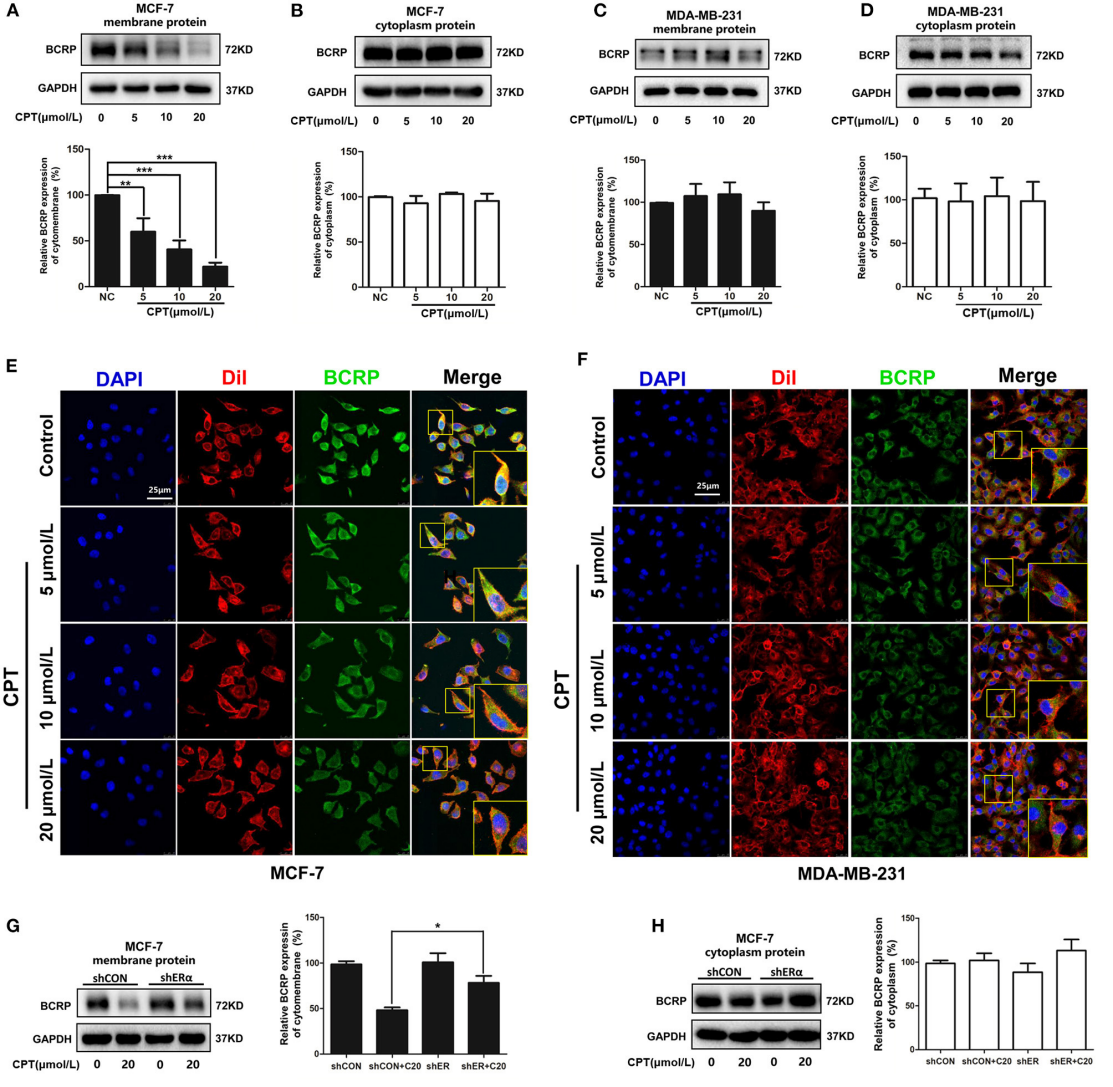
Cryptotanshinone (CPT) reduces the expression and localization of breast cancer resistance protein (BCRP) on the cell membrane of MCF-7 cells. Western blot analysis of BCRP in the cell membrane and cytoplasm of MCF-7 **(A,B)** and MDA-MB-231 cells **(C,D)** treated with CPT for 8 h vs. negative control (NC), *n* = 3, **P < 0.01, ***P < 0.001. Immunofluorescence staining for BCRP in MCF-7 **(E)** and MDA-MB-231 cells **(F)** treated with CPT for 8 h. Cell membrane was labeled with DiI. Scale bar, 25 μm. **(G,H)** MCF-7 cells with shCON or with shERα, were, respectively, treated with or without CPT (20 μmol/L) for 8 h, and then cell membrane protein and cytoplasmic protein were extracted to analyze the levels of BCRP. *n* = 3, *P < 0.05.

BCRP is a semi-transporter on the cell membrane and functions as an oligomer (21). Next, we examined whether CPT affected the level and oligomerization of BCRP on the cell membrane. Our fractionation experiment showed that CPT treatment significantly reduced the level of BCRP on the cell membrane rather than in the cytoplasm in MCF-7 cells (Figures 3A,B). However, CPT treatment had no effect on the level of BCRP on the cell membrane or in the cytoplasm of MDA-MB-231 cells (Figures 3C,D). The results indicated an inhibitory effect of CPT on the function of BCRP in ER-positive breast cancer cells. This was further verified by our laser confocal scanning microscope (LCSM) experiment. As seen in Figure 3E, when MCF-7 cells were treated with high concentrations of CPT (10 and 20 μmol/L), the enrichment of FITC-BCRP on the cell membrane dyed with Dil was obviously inhibited. But this phenomenon was not observed in MDA-MB-231 cells (Figure 3F). Additionally, we confirmed the role of ERα during this process again. Compared with membrane BCRP expression in shCON MCF-7 cells, CPT inhibition of BCRP was partially enriched in ERα-silenced MCF-7 cells (Figure 3G). Cytoplasm BCRP had no significant change (Figure 3H). Collectively, our observations support the notion that CPT inhibits the efflux function of BCRP through reducing its expression on breast cancer cell membrane, which is dependent on the presence of ERα in the cells.

### Cryptotanshinone Inhibits the Oligomer Formation of Breast Cancer Resistance Protein on the Cell Membrane

**Figure 4:**
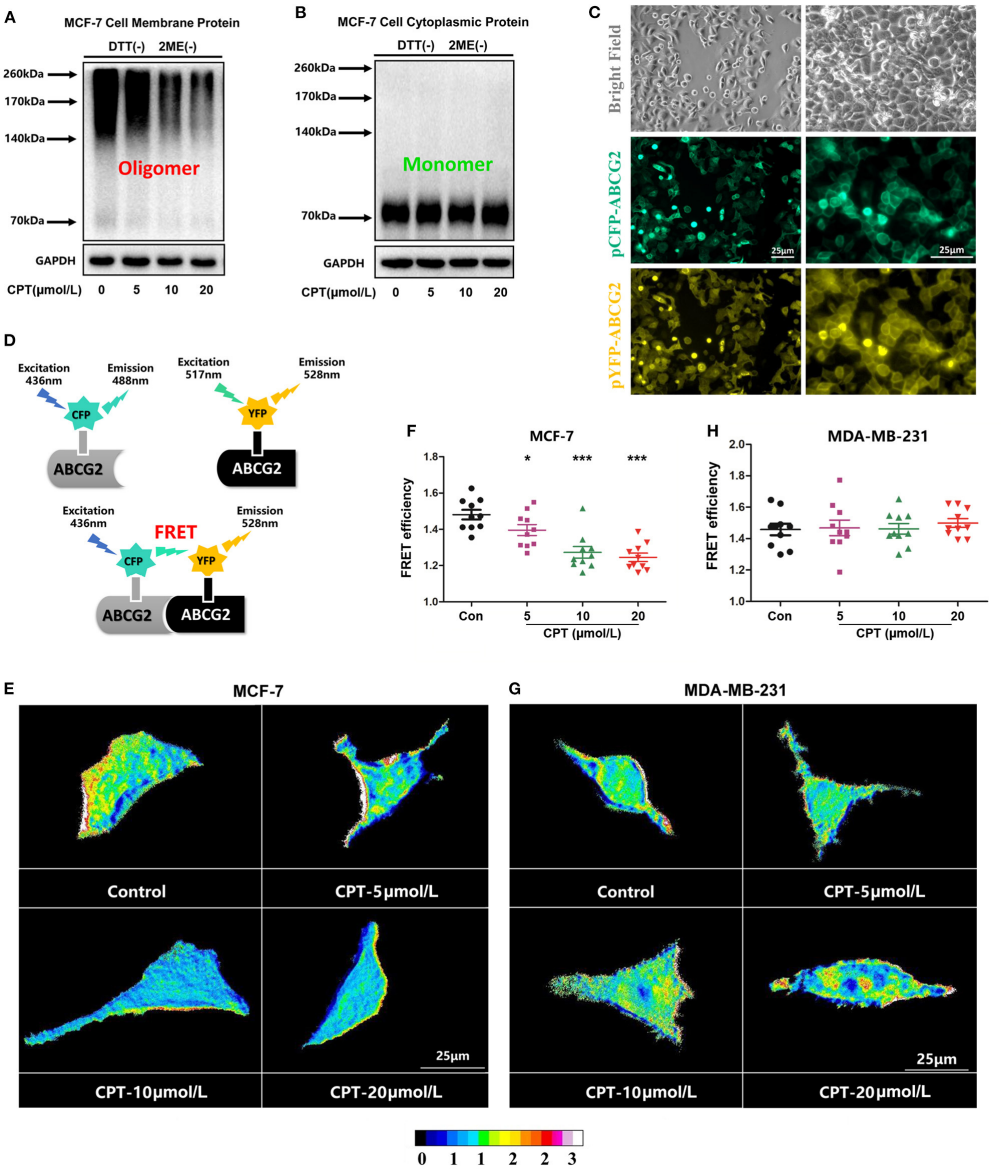
Cryptotanshinone (CPT) inhibits breast cancer resistance protein (BCRP) oligomer formation in MCF-7 cells. **(A,B)** MCF-7 cells were treated with or without CPT (20 μmol/L) for 8 h. The membrane protein or cytoplasmic protein was extracted to detect the oligomer formation of BCRP under a non-reducing condition. **(C)** MCF-7 cells were transfected with pCFP-ABCG2 and pYFP-ABCG2 plasmids, respectively, for 24 h. The expression of BCRP on the cell membrane was observed under a microscope (200 × and 400 × magnification). **(D)** Schematic diagram of fluorescence resonance energy transfer (FRET) experiment; pCFP and pYFP are the donor and acceptor of fluorescence energy, respectively. When their distance reached within 10 nm, the FRET phenomenon would happen. FRET was applied to analyze the oligomer formation of BCRP in MCF-7 cells **(E,G)** and MDA-MB-231 cells **(F,H)** treated with CPT in the state of living cells. Images are shown as 16 colors map, and the color represents the degree of FRET efficiency. The quantitated data of FRET are vs. control, *n* = 10, *P < 0.05, ***P < 0.001.

Next, we evaluated the effect of CPT on the oligomerization of BCRP using the assay of non-reducing gradient gel electrophoresis. As shown in Figure 4A, BCRP on the membrane of MCF-7 was primarily in the form of dimers and oligomers (molecular weight over 140 kDa). Oligomer formation was significantly inhibited by treatment with CPT (10 and 20 μmol/L). BCRP was also detected in the cytoplasm of MCF-7 cells, but its main form is a non-functional monomer with a molecular weight of 70 kDa (Figure 4B). Besides, after transfection, pCFP-ABCG2 and pYFP-ABCG2 were successfully expressed on the cell membrane of MCF-7 or MDA-MB-231 cells (Figure 4C). Our FRET assay revealed that CPT inhibited the BCRP oligomer formation on the cell membrane (Figure 4D), which was consistent with the observation from the non-reducing electrophoresis. CPT was able to interfere with BCRP oligomer formation in MCF-7 cells (Figures 4E,F), but not in MDA-MB-231 cells (Figures 4G,H). These data indicate that CPT inhibits the oligomer formation of BCRP on the cell membrane, thus impeding its efflux function.

### Cryptotanshinone Inhibits Breast Cancer Resistance Protein in Doxorubicin-Resistant Breast Cancer Cells Independent of Estrogen Receptor α

**Figure 5:**
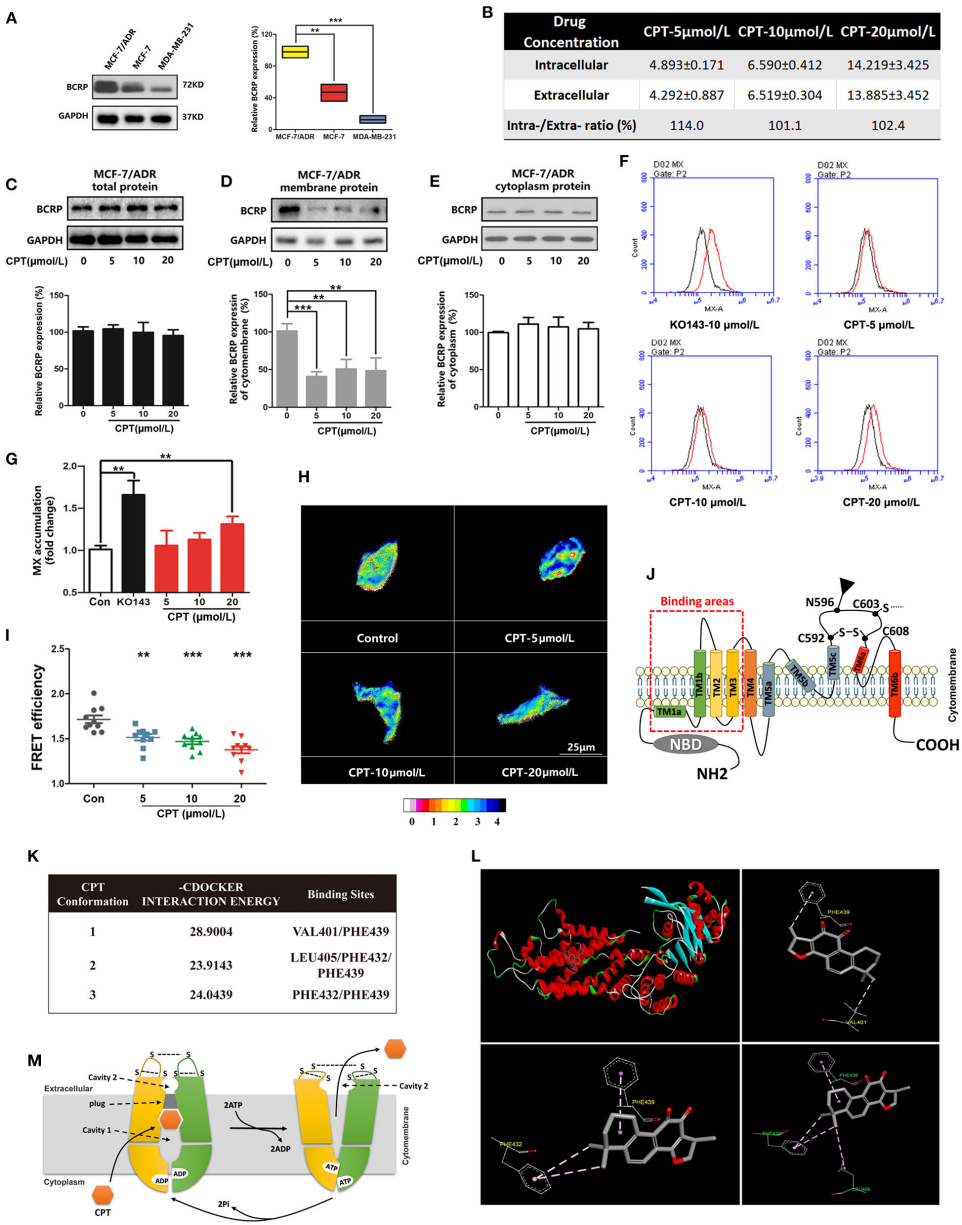
Cryptotanshinone (CPT) inhibits membrane expression and function of breast cancer resistance protein (BCRP) in MCF-7/ADR cells. **(A)** Western blot analysis of BCRP in different breast cancer cells. *n* = 3, **P < 0.01. **(B)** High-performance liquid chromatography (HPLC) analysis of the intracellular and extracellular concentrations (μmol/L) of CPT in MCF-7/ADR cells treated with CPT for 8 h. The data are presented as mean ± SD. Western blot analysis of the BCRP total protein **(C)**, membrane protein **(D)**, cytoplasm protein **(E)** expression in MCF-7/ADR cells treated with CPT for 8 h. **(F)** Mitoxantrone (MX) fluorescence accumulation was detected by flow cytometry in MCF-7/ADR cells treated with CPT for 8 h, and **(G)** the quantitated data were vs. control, *n* = 3, **P < 0.01. **(H)** Fluorescence resonance energy transfer (FRET) analysis of the oligomer formation of BCRP in MCF-7/ADR cells treated with CPT in the state of living cells. Images are shown as 16 colors map, and the color represents the degree of FRET efficiency. **(I)** The quantitated data were vs. control, *n* = 10, **P < 0.01, ***P < 0.001. **(J)** Schematic diagram of BCRP structure; TM1, TM2, TM3 are substrate-binding regions. **(K)** The molecular docking analysis about the sites of CPT docking with BCRP and their interaction energy. **(L)** The 3D structure of CPT docking with BCRP substrate-binding pocket, and three stable conformations and binding sites are listed. **(M)** The whole process of BCRP transporting substrates.

It has been noticed that DOX-resistant breast cancer cells MCF-7/ADR are sensitive to CPT (16). As indicated in Figure 5A, a high level of BCRP expression was detected in MCF-7/ADR cells, but very little in MDA-MB-231 cells. Next, we explored whether the cytotoxic effect of CPT on MCF-7/ADR cells is also dependent on inhibition of BCRP. Firstly, HPLC analysis showed that the concentration of intracellular CPT was nearly equivalent to that of extracellular CPT in MCF-7/ADR cells (Figure 5B). This result suggests that the transporters are involved in the inhibition of MCF-7/ADR cell proliferation by CPT. Despite the high expression of P-gp and MRP1 in MCF-7/ADR cells, CPT did not apparently alter the protein levels of P-gp and MRP1 either on the cell membrane or in the cytoplasm, as detected by Western blotting (Supplementary Figures 1A–D). In line with this, the flow cytometric assay indicated that CPT did not affect the efflux functions of P-gp and MRP1 (Supplementary Figures 1E–G). So, after excluding P-gp and MRP1 proteins, we focused on BCRP. Further experiments showed that CPT had no significant effect on the total cellular protein expression of BCRP in MCF-7/ADR cells (Figure 5C) but strongly inhibited the membrane protein expression of BCRP even at a low concentration (5 μmol/L) (Figure 5D). CPT treatment increased the BCRP content in the cytoplasm to some extent (Figure 5E). Similarly, the flow cytometry results showed that CPT inhibited the efflux function of BCRP in MCF-7/ADR cells, especially at a high concentration (20 μmol/L) (Figures 5F,G). FRET experiments also demonstrated that CPT inhibited the oligomer formation of BCRP in MCF-7/ADR cells (Figures 5H,I). It is worth noting that MCF-7/ADR cells have undergone many changes in morphology, characteristics, and functions compared with parental MCF-7 cells, especially MCF-7/ADR cells are ERα-negative. This suggests that CPT is also able to inhibit the function of BCRP in ERα-negative cells with high expression of BCRP.

BCRP consists of an NBD and a TMD (7), as well as substrate-binding regions TM1-3 (Figure 5J). To further determine whether CPT can be recognized by BCRP and extracellularly pumped out, molecular docking was used to dock MX, a substrate of BCRP, with the 3D structure of BCRP (Supplementary Figures 2A,B). After confirming the drug-binding pocket, the 3D structure of CPT was embedded for docking (Figures 5K,L). As shown in Figure 5K and Supplementary Figure 2A, through the intermolecular force comparison and calculation, the common binding sites were found to be VAL401, PHE439, LEU405, PHE432, and PHE439. The results indicated that CPT can be recognized by BCRP and pumped out of cells. The whole transport simulation process is shown in Figure 5M. When the substrate is bound into the cavity 1, the conformation of the protein changes with the transition from inward open to outward open. NDB combines with ATP hydrolysis to provide energy for conformational changes in the protein, while the substrate is transferred into the cavity 2, which has a relatively weak binding force, and the drug can finally be pumped out. Taken together, CPT can bind to BCRP and inhibit its efflux function, leading to an accumulation of CPT in MCF-7 cells.

### Cryptotanshinone Enhances the Sensitivity of Cancer Cells to Chemotherapeutic Drugs

**Figure 6:**
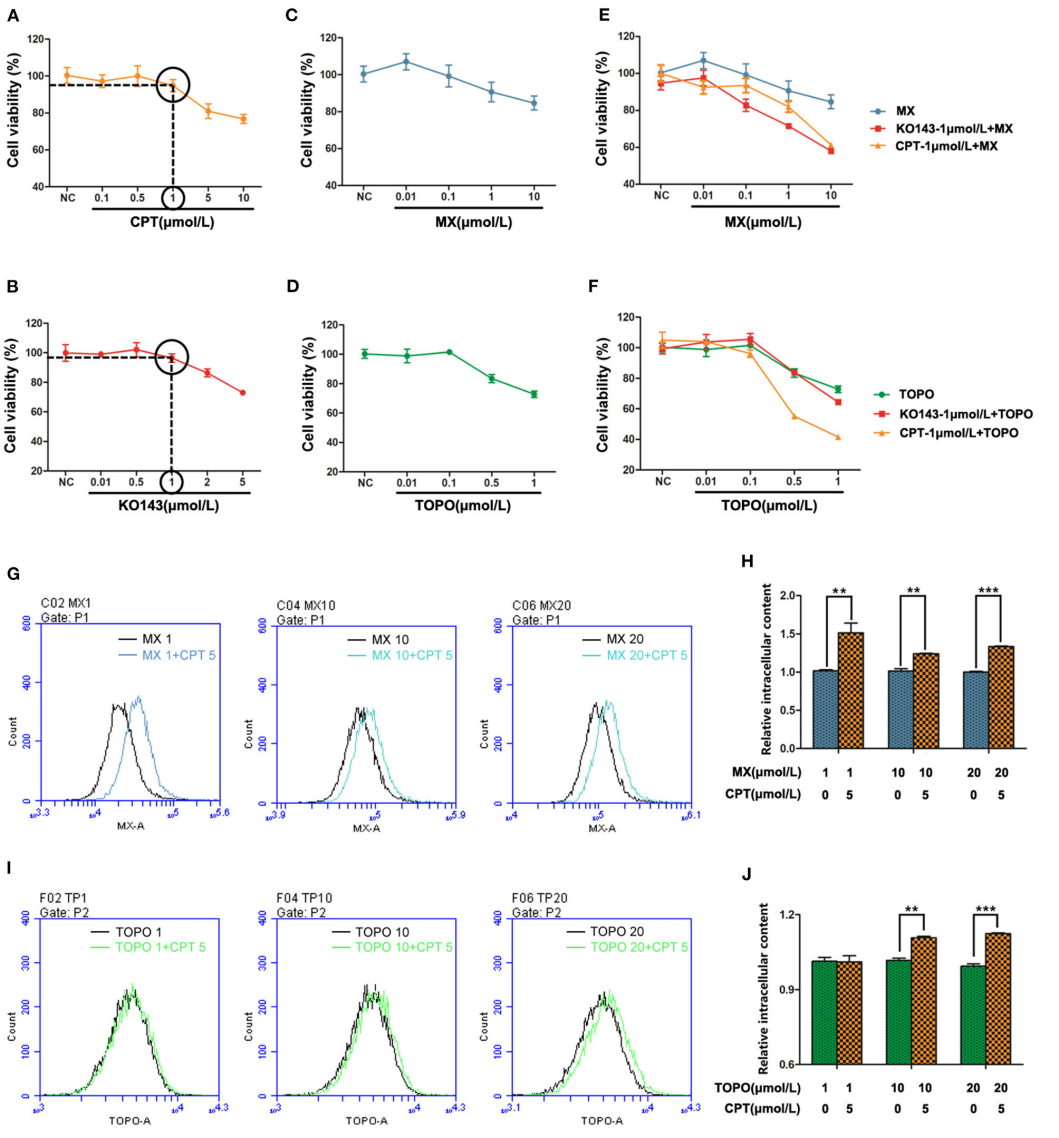
Cryptotanshinone (CPT) synergizes with breast cancer resistance protein (BCRP) efflux anticancer drugs in cancer cells. MCF-7/ADR cells were, respectively, treated with CPT **(A)** or Ko143 **(B)** for 72 h to screen the highest concentration that does not significantly affect cell proliferation. MCF-7/ADR cells were treated with mitoxantrone (MX) **(C)** and topotecan (TOPO) **(D)** for 72 h to test cell viability. **(E)** Cell proliferation of MCF-7/ADR cells treated with MX and Ko143 1 μmol/L + MX and CPT-1 μmol/L + MX for 72 h. **(F)** Cell proliferation of MCF-7/ADR cells treated with TOPO and Ko143 1 μmol/L + TOPO and CPT 1 μmol/L + TOPO for 72 h. MX **(G)** and TOPO **(H)** fluorescence accumulation was detected by flow cytometry in MCF-7/ADR cells, respectively, treated with CPT + MX or CPT + TOPO for 8 h, and the fluorescence intensity reflects the relative content of intracellular drugs. **(I)** Comparison of intracellular MX content in CPT + MX group and MX alone group in MCF-7/ADR cells for 8 h. Results were vs. MX alone, respectively, *n* = 3, **P < 0.01, ***P < 0.001. **(J)** Comparison of intracellular TOPO content in CPT + TOPO group and TOPO alone group in MCF-7/ADR cells for 8 h. Results were vs. TOPO alone, respectively, *n* = 3, **P < 0.01, ***P < 0.001.

Based on the above results, we reasoned that CPT may be synergistic with BCRP efflux anticancer drugs. To test this, we selected two of the most classic BCRP efflux drugs, MX and TOPO (22). At first, we screened the concentration of CPT and the positive control drug Ko143. Then, we carried out a 72-h co-incubation experiment at a concentration of 1 μmol/L, which had no significant effect on the proliferation of MCF-7/ADR cells (Figures 6A,B). Compared with the single treatment with MX (Figure 6C) or TOPO (Figure 6D), the co-treatment with CPT significantly attenuated drug resistance at higher doses (MX 10 μmol/L, TOPO 0.5 μmol/L), and the inhibition rate of proliferation was under 50%, close to the co-treatment with Ko143 (Figures 6E,F). And the IC_50_ of CPT with MX is about 26 times of that of MX alone; CPT with TOPO is about 16 times of that of TOPO treatment alone. Meanwhile, subsequent flow cytometry showed that when MCF-7/ADR cells were co-treated with CPT and MX, the accumulation of MX increased in the cells, i.e., the functional inhibition of BCRP by CPT made the drug efflux reduced (Figures 6G,H). Similar results were observed in the co-treatment with CPT and TOPO (Figures 6I,J). The findings indicate that CPT is able to enhance the sensitivity of cancer cells to chemotherapeutic agents that can be pumped out by BCRP, reversing the MDR.

## Discussion

The role of ERα in breast cancer is well-understood. ER expression is a critical factor for hormonal therapy and also considered as a prognostic marker. Overall, about 75% of breast cancer patients are ER-positive and treated with anti-estrogen drugs, such as the selective estrogen receptor modulator tamoxifen. However, a part of ERα-positive breast cancers can become resistant to hormone therapy partly due to loss of ERα expression, so a majority of these patients may suffer a relapse in 5 years (23). Furthermore, many researchers have also found that some chemotherapeutic agents may be less effective in ERα-positive breast cancer patients than ERα-negative ones (24). These bidirectional results imply the complex role of ERα in the resistant breast cancer.

Generally, breast cancer may develop MDR. Several factors including ABC transporters (5), mutations of targeted oncogenes, survived cancer stem cells (CSCs) (25), and activated cell growth factors are possibly involved in MDR. Especially BCRP, one type of the ABC transporters is an important factor controlling the breast cancer MDR. Indeed, the relationship between ERα and BCRP expression has been investigated (26). The estrogen response element (ERE) and progesterone response element (PRE) exist in the promoter region of BCRP (27). The excessive transcriptional expression of this type of response element may play a major role in the development of breast cancer MDR (11). The MDR is a multi-factor, multi-way, multi-stage, and comprehensive process (28). This study focused on BCRP primarily and expected to look for new applications of CPT to reverse breast cancer MDR.

In the previous research, we have proven that CPT is not a selective estrogen receptor inhibitor, though it can bind ERα and produce tamoxifen-like effects on cancers (16). More importantly, CPT inhibits DOX-resistant MCF-7/ADR cells, although it can activate MAPK and AKT, suggesting that CPT's overcoming the resistance is not through suppressing the activation of MAPK (29) and AKT (30) but by targeting other factors. So we studied if BCRP is the key molecule targeted by CPT. At present, numerous BCRP inhibitors including highly selective inhibitors and non-selective inhibitors have been identified, and some highly selective inhibitors should be prospectively applied in the clinic for reversing the MDR (31). However, the molecular mechanism of BCRP inhibition is complex. Some compounds inhibit BCRP through inhibiting its ATPase activity, such as FTC, Ko134, and Ko143, while others as BCRP substrates can bind to BCRP and competitively suppress the transport function of BCRP (9, 31). In this study, we found that CPT was able to interfere with the oligomer formation of BCRP on the cell membrane, thereby impeding its efflux function. On the one hand, in ERα-positive MCF-7 cells, inhibition of BCRP oligomer formation was dependent on the status of ERα, as downregulation of ERα attenuated CPT-induced accumulation of MX in the cells. How ERα mediates this process is unclear. On the other hand, in the ERα-negative MCF-7/ADR cells with a high expression of BCRP, the expression and oligomer formation of BCRP on the cell membrane were both inhibited by CPT. However, in the ERα-negative MDA-MB-231 cells with low expression of BCRP, CPT did not affect cell proliferation. These data suggest that CPT reversing resistance of breast cancer is dependent on the expression level of BCRP: the higher expression of BCRP, the stronger inhibitory effect of CPT on breast cancer cells. Although CPT is an anti-estrogen compound, it can directly bind BCRP and block its efflux function. It has been observed that breast cancer cells can switch between ERα and ErbB signaling to induce resistance, and combined inhibition of the two pathways can postpone the development of resistance (32). From MCF-7 to MCF-7/ADR, ERα is nearly undetectable but BCRP is overexpressed. Together, these observations suggest that there are two modes of CPT inhibition of BCRP: ERα-dependent and ERα-independent. CPT switches the targets between ERα and BCRP to attenuate MDR.

**Figure 7:**
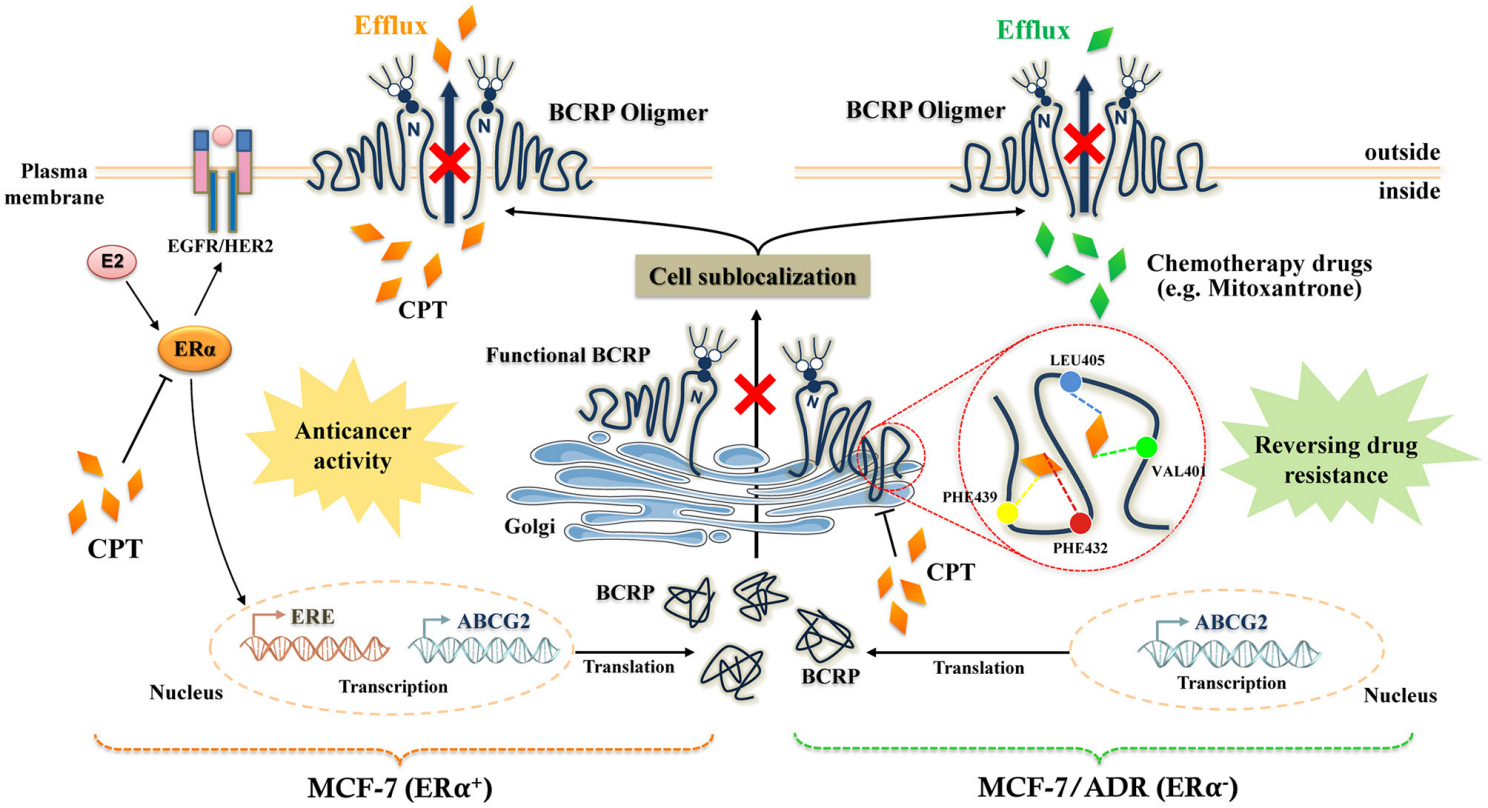
The molecular mechanistic diagram of cryptotanshinone (CPT) reversing the multidrug resistance.

Increasing evidence indicates that a drug designed for individual molecular target is generally hard to conquer multigenic diseases such as cancer, diabetes, hypertension, and neural diseases (33, 34). So combined drugs that simultaneously affect multiple targets are more beneficial to control complex disease and reverse drug resistance. If a compound could target multiple proteins under various cell circumstances, the occurrence of resistance would also be decreased to minimal extent. We believed that CPT is a multi-target compound, since it has been shown to target a number of molecules such as STAT3 (12), AMPK (15), MAPK (14), ER (16), and nuclear factor erythroid-2 related factor 2 (NRF2) (35). Though different action mechanisms have been proposed for CPT, our findings indicate that ER and BCRP can be structurally bound with CPT. Figure 7 illustrates the molecular process of CPT inhibition of BCRP in an ERα-dependent and -independent manner.

At present, designing the multi-target molecules based on the systems biology, structural biology, and chemical informatics has become an optimal way to develop a new drug (34). CPT as a multi-target compound is a potential candidate drug, particularly considering its selective inhibition of BCRP in ERα-negative breast cancer. Our observation that CPT is synergistic with anticancer drugs further highlights the great potential in clinical applications. Although the inhibitory activity of CPT is not dominant compared to some current BCRP inhibitors, CPT, as a lead natural compound, after appropriate optimization of the structure, may be developed into a highly effective and low-toxicity BCRP-specific inhibitor to serve clinical therapy.

## Conclusions

CPT is a novel natural BCRP inhibitor. It directly binds to BCRP and interferes with the oligomer formation of BCRP on the cell membrane, thereby blocking the efflux function of BCRP. In the ERα-positive cells, the inhibitory effect of CPT on BCRP is dependent on the expression level of BCRP. However, in the ERα-negative DOX-resistant breast cancer cells highly expressing BCRP, CPT can directly inhibit BCRP efflux function. So, CPT inhibits BCRP in an ERα-dependent and -independent manner. Our findings suggest that CPT has great potential to be explored for treatment of breast cancer with a high expression of BCRP regardless of the status of ERα.

## Data Availability Statement

The raw data supporting the conclusions of this article will be made available by the authors, without undue reservation.

## Author Contributions

WC and YL provided the study design and supervision and wrote the paper. WN and HF performed the experiments and analyses. SW and SH assisted with molecular analysis and interpretation. XZ assisted with data analysis and statistic. FX, YW, XL, and AW provided special reagents and/or helped in analyzing the experiments. All authors read and approved the final manuscript.

## Conflict of Interest

The authors declare that the research was conducted in the absence of any commercial or financial relationships that could be construed as a potential conflict of interest.

## Supplementary Material

Supplementary Figure 1 Effect of CPT on P-gp and MRP1 protein expression and efflux function in MCF-7/ADR cells. Western blot analysis of P-gp and MRP1 cell membrane protein expression **(A)** and cytoplasmic protein expression **(C)** in MCF-7/ADR cells treated with CPT for 8 h, and the corresponding semi-quantitation analyses are shown in **(B,D)**. RH123 **(E)** and DOX **(F)** fluorescence accumulation in MCF-7/ADR cells treated with CPT for 8 h was, respectively, detected by flow cytometry, and the fluorescence intensity represents the activity of P-gp and DOX efflux. RH123 and DOX were, respectively, the substrates of P-gp and MRP1. **(G)** The quantitated data of **(E)** and **(F)**.

Supplementary Figure 2 The situation of mitoxantrone binding to BCRP. **(A,B)** The 3D structure of BCRP-specific substrate mitoxantrone (MX) is docked with BCRP to find substrate binding pocket, and three stable conformations, binding sites, and interaction energy are listed.

Supplementary Figure 3 **(A)** The chemical structure of cryptotanshinone. **(B)** The HPLC chemoprofile of cryptotanshinone.

Supplementary Table 1 Primers used for determination of ABCG2 mRNA expression levels in MCF-7 cells and MDA-MB-231 cells.

## Abbreviations

CPT: cryptotanshinone
BCRP: breast cancer resistance protein
ERα: estrogen receptor α
MDR: multidrug resistance
ABC: ATP-binding cassette
MRP1: multidrug resistance-associated protein
NBD: nucleotide-binding domain
TMD: transmembrane domain
MX: mitoxantrone
DOX: doxorubicin
TOPO: topotecan
FRET: fluorescence resonance energy transfer
STAT3: signal transducer and activator of transcription 3
AMPK: AMP-activated protein kinase
MAPK: mitogen-activated protein kinase
NRF2: nuclear factor erythroid-2 related factor 2.
