# Supplementary figures and images for: Cryptotanshinone Inhibits ERα-Dependent and -Independent BCRP Oligomer Formation to Reverse Multidrug Resistance in Breast Cancer
Source: Front Oncol. 2021 Apr 22;11:624811. doi: 10.3389/fonc.2021.624811 (PMC8100513; doi:10.3389/fonc.2021.624811)

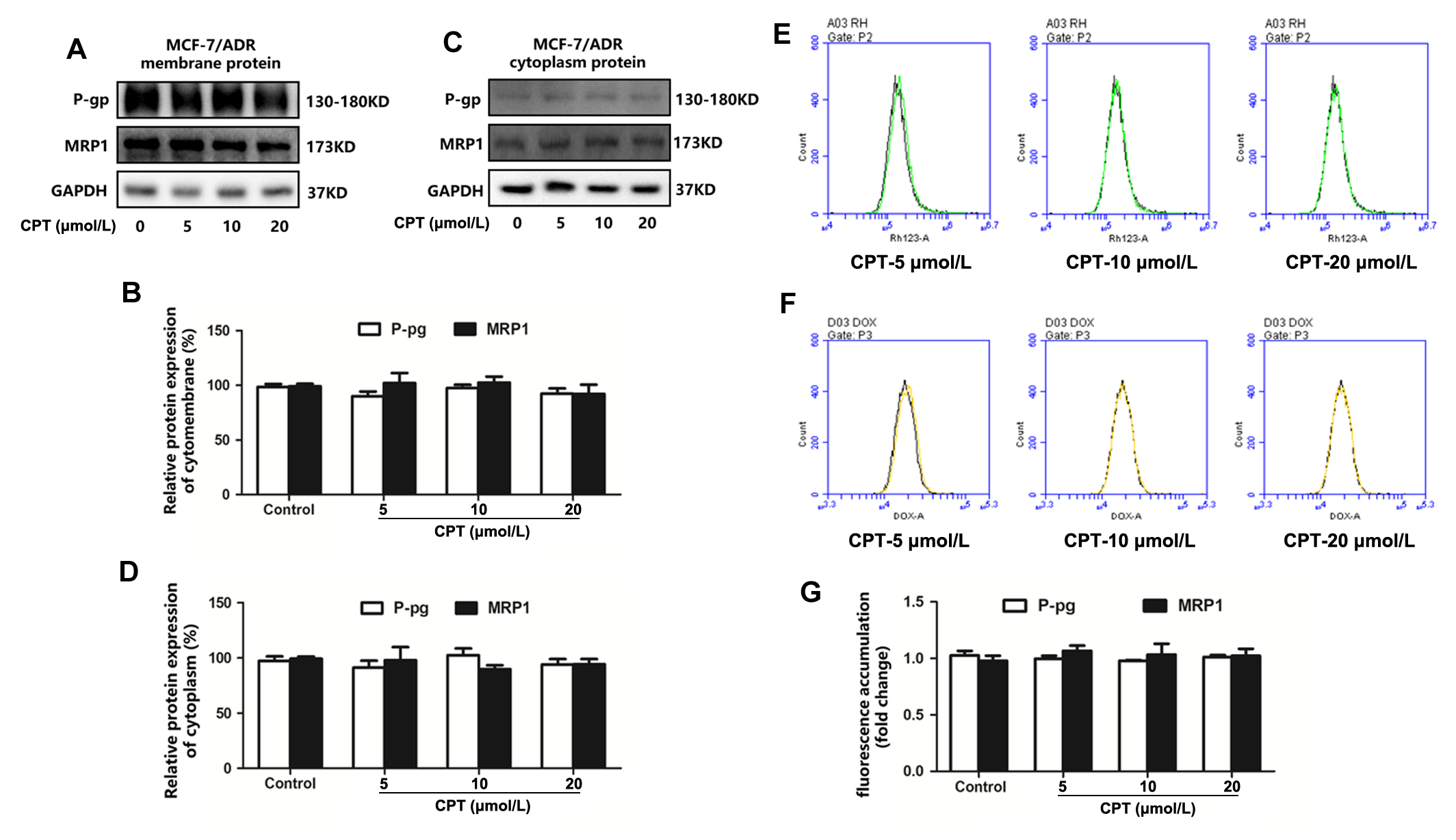

Supplement: Supplementary Figure 1 — Effect of CPT on P-gp and MRP1 protein expression and efflux function in MCF-7/ADR cells. Western blot analysis of P-gp and MRP1 cell membrane protein expression (A) and cytoplasmic protein expression (C) in MCF-7/ADR cells treated with CPT for 8 h, and the corresponding semi-quantitation analyses are shown in (B,D). RH123 (E) and DOX (F) fluorescence accumulation in MCF-7/ADR cells treated with CPT for 8 h was, respectively, detected by flow cytometry, and the fluorescence intensity represents the activity of P-gp and DOX efflux. RH123 and DOX were, respectively, the substrates of P-gp and MRP1. (G) The quantitated data of (E) and (F). [file Image_1.TIF]

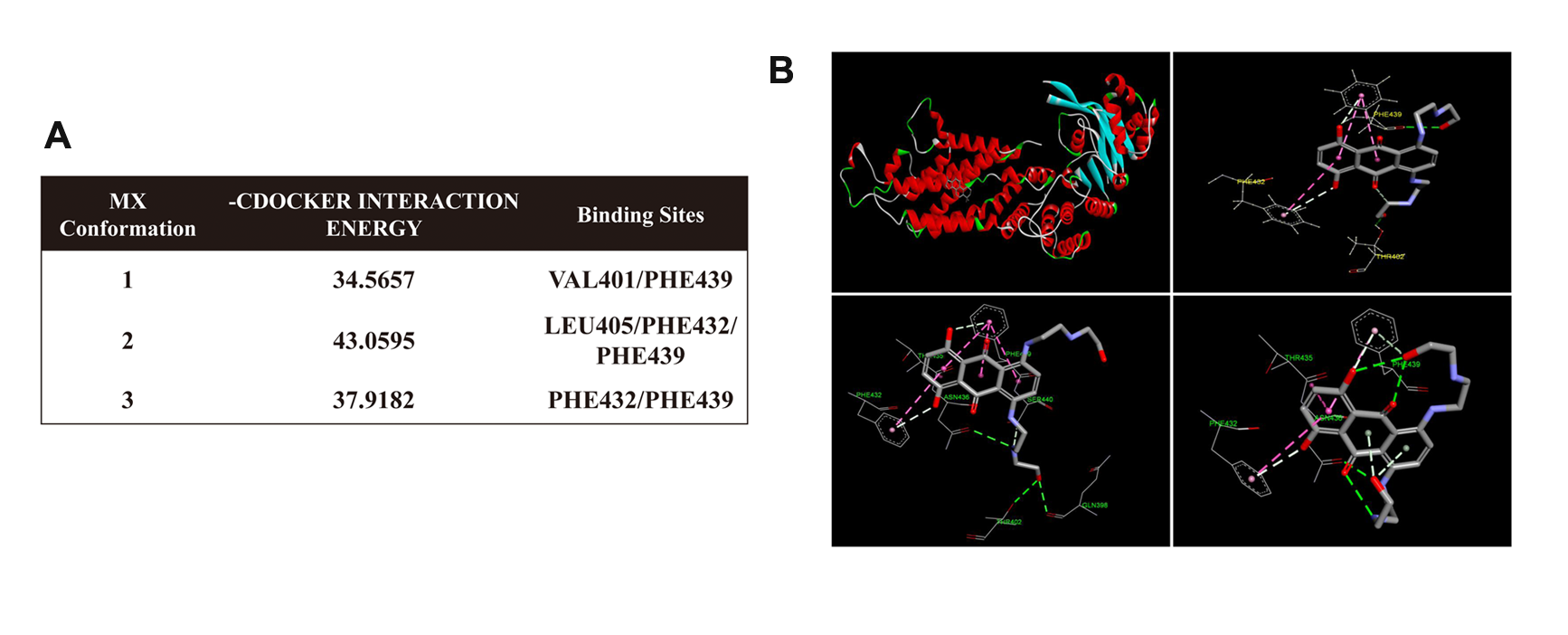

Supplement: Supplementary Figure 2 — The situation of mitoxantrone binding to BCRP. (A,B) The 3D structure of BCRP-specific substrate mitoxantrone (MX) is docked with BCRP to find substrate binding pocket, and three stable conformations, binding sites, and interaction energy are listed. [file Image_2.TIF]
