# Supplementary material for: Cryptotanshinone Inhibits ERα-Dependent and -Independent BCRP Oligomer Formation to Reverse Multidrug Resistance in Breast Cancer
Source: Front Oncol. 2021 Apr 22;11:624811. doi: 10.3389/fonc.2021.624811 (PMC8100513; doi:10.3389/fonc.2021.624811)
Supplement: Supplementary Figure 3 — (A) The chemical structure of cryptotanshinone. (B) The HPLC chemoprofile of cryptotanshinone. [file Image_3.pdf]

A

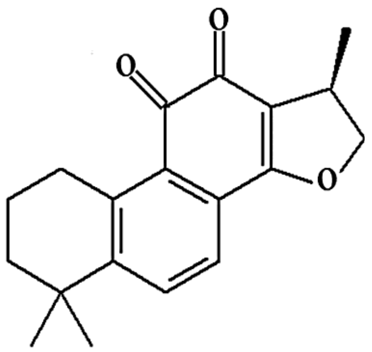

B

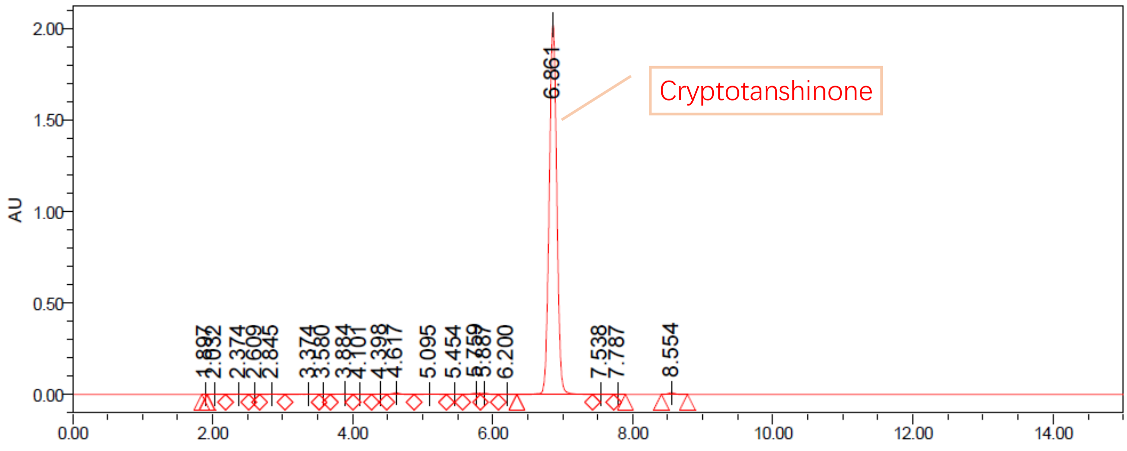

Fig.S3 A. the chemical structure of cryptotanshinone; B. the HPLC chemoprofile of cryptotanshinone.
