# Supplementary material for: Cryptotanshinone Inhibits ERα-Dependent and -Independent BCRP Oligomer Formation to Reverse Multidrug Resistance in Breast Cancer
Source: Front Oncol. 2021 Apr 22;11:624811. doi: 10.3389/fonc.2021.624811 (PMC8100513; doi:10.3389/fonc.2021.624811)
Supplement: Supplementary Table 1 — Primers used for determination of ABCG2 mRNA expression levels in MCF-7 cells and MDA-MB-231 cells. [file Table_1.docx]

**Table legends**

Table.S1 Primers used for determination of ABCG2 mRNA expression levels in MCF-7 cells and MDA-MB-231 cells.

| Gene | Forward sequence | Reverse Sequence |
| --- | --- | --- |
| ABCG2 | 5’-CAGGTGGAGGCAAATCTTCGT-3’ | 5’-ACCCTGTTAATCCGTTCGTTTT-3’ |
| GAPDH | 5’-GGAGCGAGATCCCTCCAAAAT-3’ | 5’-GGCTGTTGTCATACTTCTCATGG-3’ |
